# Full-Length Venom Protein cDNA Sequences from Venom-Derived mRNA: Exploring Compositional Variation and Adaptive Multigene Evolution

**DOI:** 10.1371/journal.pntd.0004587

**Published:** 2016-06-09

**Authors:** Cassandra M. Modahl, Stephen P. Mackessy

**Affiliations:** School of Biological Sciences, University of Northern Colorado, Greeley, Colorado, United States of America; Instituto de Biomedicina de Valencia, UNITED STATES

## Abstract

Envenomation of humans by snakes is a complex and continuously evolving medical emergency, and treatment is made that much more difficult by the diverse biochemical composition of many venoms. Venomous snakes and their venoms also provide models for the study of molecular evolutionary processes leading to adaptation and genotype-phenotype relationships. To compare venom complexity and protein sequences, venom gland transcriptomes are assembled, which usually requires the sacrifice of snakes for tissue. However, toxin transcripts are also present in venoms, offering the possibility of obtaining cDNA sequences directly from venom. This study provides evidence that unknown full-length venom protein transcripts can be obtained from the venoms of multiple species from all major venomous snake families. These unknown venom protein cDNAs are obtained by the use of primers designed from conserved signal peptide sequences within each venom protein superfamily. This technique was used to assemble a partial venom gland transcriptome for the Middle American Rattlesnake (*Crotalus simus tzabcan*) by amplifying sequences for phospholipases A_2_, serine proteases, C-lectins, and metalloproteinases from within venom. Phospholipase A_2_ sequences were also recovered from the venoms of several rattlesnakes and an elapid snake (*Pseudechis porphyriacus*), and three-finger toxin sequences were recovered from multiple rear-fanged snake species, demonstrating that the three major clades of advanced snakes (Elapidae, Viperidae, Colubridae) have stable mRNA present in their venoms. These cDNA sequences from venom were then used to explore potential activities derived from protein sequence similarities and evolutionary histories within these large multigene superfamilies. Venom-derived sequences can also be used to aid in characterizing venoms that lack proteomic profiles and identify sequence characteristics indicating specific envenomation profiles. This approach, requiring only venom, provides access to cDNA sequences in the absence of living specimens, even from commercial venom sources, to evaluate important regional differences in venom composition and to study snake venom protein evolution.

## Author Summary

This work demonstrates that full-length venom protein messenger RNAs are present in secreted venoms and can be used to acquire full-length protein sequences of toxins from both front-fanged (Elapidae, Viperidae) and rear-fanged (Colubridae) snake venoms, eliminating the need to use venom glands. Full-length transcripts were obtained from venom samples that were fresh, newly lyophilized, old, field desiccated or commercially prepared, representing a significant advance over previous attempts which produced only partial sequence transcripts. Transcripts for all major venom protein families (metalloproteinases, serine proteases, C-type lectins, phospholipases A_2_ and three-finger toxins) responsible for clinically significant snakebite symptoms were obtained from venoms. These sequences aid in the identification and characterization of venom proteome profiles, allowing for the identification of peptide sequences, specific isoforms, and novel venom proteins. The application of this technique will help to provide venom protein sequences for many snake species, including understudied rear-fanged snakes. Venom protein transcripts offer important insights into potential snakebite envenomation profiles and the molecular evolution of venom protein multigene families. By requiring only venom to obtain venom protein cDNAs, the approach detailed here will provide access to cDNA-based protein sequences from commercial and other venom sources, facilitating study of snake venom protein composition and evolution.

## Introduction

The evolution of venoms among the advanced colubroid snakes has had tremendous adaptive significance and has allowed this clade to diversify rapidly and occupy a diversity of niches globally [1]. Snake venoms are complex glandular secretions that may contain 2–100+ protein/peptide components with a myriad of biological activities, ranging from potent neurotoxins to rapid-acting myotoxins to hydrolytic enzymes [2]. These toxins are synthesized and stored in a cephalic venom gland which allows immediate deployment as a chemical weapon, also necessitating intricate storage and protective mechanisms [3]. Venoms likely allowed a transition from mechanical capture and processing of prey to one dependent on chemical means [4], and during the approximately 100 million year history of snakes [5], a diversity of biochemical compositional “strategies” have evolved [6, 7]. Resulting venom phenotypes can therefore be significantly different, even among closely related species [2], and these different phenotypes are often correlated with dietary variables or foraging strategies [8–10]. Determining detailed venom composition among differing lineages of snakes can provide important connections linking phenotypic variation to specific selective pressures, and linking venom composition to snakebite envenomation effects.

The application of transcriptomic methods has provided insight into venom protein post-transcriptional regulation, as well as documenting isoform diversity and molecular evolutionary trends within large multigene venom protein superfamilies [11–15]. Venom gland transcriptomics has evolved from the generation of ESTs (expressed sequence tags) [16–22] to more comprehensive next generation sequencing (454 pyrosequencing or Illumina) of total venom gland cDNA (complementary DNA) [23–28]. However, these methods both currently rely on venom gland tissue to obtain venom protein cDNAs, requiring access to venomous snake tissues and animal euthanasia. The ability to acquire venom protein cDNA sequences from venom has been documented [29–32], but this source has not been fully exploited because mRNA yields have been highly variable and very low, and cDNA amplification has previously focused only on known venom protein transcripts and only partial transcripts were amplified. Extracellular messenger RNA has been demonstrated to be unusually stable, for at least several years within lyophilized venom [30]. This alternative source to obtain venom protein cDNAs is a less destructive method because sacrifice of animals is avoided. It also increases the availability of venom protein cDNA sequences to researchers that have limited access to venom gland tissues, as in the case of rare or difficult to acquire snake species, or due to limitations on animal euthanasia protocols. Further, this approach provides the opportunity to generate both venom transcriptomic and proteomic profiles, essentially a genotype-phenotype map, using only the same venom sample from one individual.

The standardized venomics approach of characterizing venoms by separating venom components by HPLC (high performance liquid chromatography), followed by trypsin digestion and tandem mass spectrometry, primarily relies on protein identification from databases such as MASCOT [33]. Mass spectral matching to determine peptide sequences is an efficient and less expensive alternative to N-terminal sequencing (Edman degradation). An advantage to this venomic approach is that it is at least semi-quantitative, allowing inter- and intrapopulational variation in amounts of specific proteins to be estimated. However, many venom proteins, in particular those from rear-fanged venomous snakes, are not present within current databases or are poorly represented, making it difficult to use this methodology to characterize these venoms [9, 33, 34]. The incorporation of transcriptomics into venom proteomics has resulted in venom protein-locus resolution and has been labeled next generation snake venomics [18, 23, 28, 34]. Species-specific venom gland transcriptomes aid in the identification and characterization of venom profiles by providing custom databases for tandem mass spectrometry (MS/MS) spectra matching, allowing for the identification of additional peptide sequences, specific isoforms, and novel venom proteins [16, 24, 28, 34, 35]. This study provides support for an approach to obtain species-specific venom protein transcript sequences, including those from rear-fanged venomous snakes, using relatively little starting material (2 mg of lyophilized venom or 100 μl of fresh venom) that does not require venom gland tissue. cDNA derived from this method has great potential to fill gaps within databases and to aid in the characterization of understudied snake venoms.

Acquiring full-length venom protein sequences can also help to identify protein characteristics indicative of serious envenomation profiles, because venom toxins commonly utilize conserved structural folds but produce diverse pharmacological activities. For example, many venoms contain enzymatic phospholipase A_2_s (PLA_2_s) of low toxicity [36, 37]; some contain PLA_2_s with potent myotoxic activity, and a limited number contain neurotoxic PLA_2_s (crotoxin-like complexes or other asparagine-6 containing PLA_2_ sequences). Current methods for examining venom protein pharmacological activity can be labor intensive, requiring multiple protein purification steps and functional assays. Phospholipase A_2_ functionality based on clustering with other PLA_2_s sharing similar sequence has been demonstrated to be a potential *in silico* alternative for predicting specific PLA_2_ activity [37]. Knowledge of sequences and sequence similarities to others with noted activity and that are currently promising drug leads can help guide new drug exploration or even help to identify unique characteristics of prey-specific toxins that allow them to specifically target selected prey receptors, as in the case of rear-fanged snake venom three-finger toxins (3FTxs).

Rear-fanged snake venoms have not been as well-studied as front-fanged snake venoms, largely due to the difficulties extracting venoms from these snakes and the fact that the large majority of envenomations from these snakes are not life threatening [38–41]. However, rear-fanged snake venoms potentially contain proteins that could serve as novel pharmaceutical drug leads or in other applications, such as proteolytic enzymes for protein fragmentation for mass spectrometry [42, 43]. Rear-fanged snake venoms have also demonstrated unique evolutionary trajectories, including the presence of the only prey-specific toxins yet identified within snake venoms [8, 44, 45].

The aim of this study was to obtain cDNA sequences of abundant venom proteins to predict protein activities or envenomation symptomology. From the rattlesnake PLA_2_ transcripts identified, sequences characteristic of known viper neurotoxic PLA_2_s were observed, demonstrating the utility of using these methods to obtain predictive venom activities and envenomation profiles. In addition, this technique could be used to screen for novel sequences that could be of potential biomedical development based on predicted activities, and to explore the evolutionary trajectories in complex multigene superfamilies.

## Methods

### Snake Venoms and Reagents

Venom was collected from front-fanged vipers *Crotalus scutulatus scutulatus* (Mohave Rattlesnake; SE Arizona), *Crotalus cerastes* (Sidewinder; S Arizona), *Crotalus oreganus cerberus* (Arizona Black Rattlesnake; E Arizona), *Crotalus oreganus concolor* (Midget Faded Rattlesnake; S. Wyoming), and *Sistrurus miliarius barbouri* (Florida Pigmy Rattlesnake; central Florida) by placing an RNase Away (Thermo Fisher Scientific Inc., U.S.A.)-treated 100 μl capillary tube over each fang and gently massaging the gland; 100 μl of venom was then immediately added to 1 mL of TRIzol (Life Technologies, CA, U.S.A.). Venom from *Crotalus simus tzabcan* (Middle American Rattlesnake; Yucatán Peninsula, México) was extracted into a sterile beaker and 10 μl, 25 μl, 50 μl and 100 μl of venom were each immediately added to 1 mL of TRIzol; the remaining venom was then centrifuged (9500 x g for 5 minutes), lyophilized and stored at -20°C until used. Venom from *Crotalus molossus nigrescens* (Mexican Black-tailed Rattlesnake; Morelia, México) was collected in the field, desiccated, and stored at -20°C until used. Lyophilized venom from *Crotalus pricei* (Twin-spotted Rattlesnake; SE Arizona) was collected from a captive snake and stored frozen (-20°C) with desiccant for approximately 20 years; lyophilized *Crotalus basiliscus* (Mexican West Coast Rattlesnake; W. México) venom was purchased from the Miami Serpentarium (Lot#CB15SZ) and lyophilized venom from an elapid snake, *Pseudechis porphyriacus* (Red-bellied Black Snake; E Australia), was a gift from Venom Supplies Pty Ltd (Tanunda, South Australia).

Venoms from the rear-fanged snakes *Boiga irregularis* (Brown Treesnake, Guam), *Boiga dendrophila* (Mangrove Snake; Indonesia), *Boiga nigriceps* (Black-headed Catsnake; Indonesia), *Trimorphodon biscutatus lambda* (Sonoran Lyre Snake; Portal, AZ), and *Alsophis portoricensis* (Puerto Rican Racer; Guana Island, British Virgin Islands) were extracted using the method of Hill and Mackessy (1997) with subcutaneous injections of ketamine-HCl (20–30 mg/kg) followed by pilocarpine-HCl (6 mg/kg). Venom was collected by placing RNase Away-treated 100 μl capillary tubes over each enlarged rear maxillary tooth [46], and venom (100 μl) was then added to 1 mL of TRIzol. For the rear-fanged snakes *Boiga cynodon* (Dog-toothed Catsnake; Indonesia), *Oxybelis fulgidus* (Green Vine Snake; Central America), and *Ahaetulla prasina* (Asian Vine Snake; Indonesia), venom was collected using the same protocol without RNase Away treated capillary tubes, centrifuged (9500 x g for 5 minutes), lyophilized, and stored at -20°C until used.

The 3’ RACE System kit, PCR SuperMix High Fidelity polymerase, custom oligonucleotides, DNase I, and *Escherichia coli* DH5 α competent cells were purchased from Life Technologies, CA, U.S.A. The plasmid Quick Clean 5M Miniprep Kit was from GenScript, Inc (Piscataway Township, NJ, U.S.A), and the pGEM-T Easy Vector System and Wizard SV gel and PCR clean-up system from Promega, Inc. (Madison, WI, U.S.A.) All other reagents were purchased from Sigma (St. Louis, MO, U.S.A).

### Ethics Statement

Many of the snake venoms were collected manually from venomous snakes maintained in the University of Northern Colorado Animal Resource Facility in accordance with protocols #9204 and 1302D-SM-S-16 (evaluated and approved by the UNC IACUC) and collecting permits from state and federal agencies (Arizona Game and Fish Department #MCKSY000221 and #SP727017; Colorado Parks and Wildlife #15HP974; U.S. Fish and Wildlife Service #MA022452-0). Snakes are maintained for venoms in accordance with guidelines published by the American Society of Ichthyologists and Herpetologists [47].

### Venom RNA Isolation and cDNA Synthesis

RNA was purified from 2 mg of lyophilized venom or 100 μl of freshly collected venom that had been added to 1 mL of TRIzol following the recommended TRIzol RNA protocol: after incubation for 5 minutes, 200 μL of chloroform was added to each tube, tubes were centrifuged at 12,000 x g for 15 minutes, aqueous upper phases were transferred to new RNase-free tubes, and 500 μL 100% isopropanol added to each aqueous phase to precipitate RNA. Tubes were incubated at room temperature for 10 minutes and centrifuged at 12,000 x g for 10 minutes. Supernatant was removed and the resulting RNA pellet (not visible) washed with 1 mL 75% ethanol. Another centrifuge step at 7,500 x g for 5 minutes was performed and supernatant poured off. A -20°C overnight incubation in 300 μL 100% ethanol with 40 μL 3 M sodium acetate was then performed to increased RNA yields, and the following day tubes were centrifuged at 10,000 x g for 15 minutes, supernatant removed, and total RNA resuspended in 16 μL nuclease-free H_2_O. To evaluate the effect of different amounts of lyophilized and fresh venom on cDNA yields, 5 mg, 10 mg, and 20 mg of lyophilized venom or 10, 25, 50 or 100 μL of crude fresh venom from *C*. *s*. *tzabcan* was added to TRIzol reagent and processed as above. For rear-fanged snake venoms, extraction methods resulted in retention of significant amounts of contaminating DNA; therefore, an Amplification Grade DNase I digestion after RNA isolation was performed at room temperature for 15 minutes, followed by the addition of 1 μl 25 mM EDTA (pH 8.0) and a 15 minute 65°C incubation. cDNA synthesis from total RNA was accomplished using the 3’ RACE System following the manufacturer’s protocols. The oligo(dT) adaptor primer provided with the kit initiated reverse transcriptase cDNA synthesis and effectively selected for polyadenylated mRNAs.

### 3’RACE (Rapid Amplification of cDNA Ends)

Sense primer sequences were designed from conserved signal peptide regions for each venom protein superfamily (Table 1). To identify conserved signal peptide sequences, multiple sequence alignments within MEGA v6.06 [48] using MUSCLE [49] were performed for each venom protein superfamily, with representative sequences obtained from the NCBI (National Center for Biotechnology Information) nucleotide database. Each sense primer was used in a reaction with the 3’RACE system AUAP antisense primer 5’-GGCCACGCGTCGACTAGTAC-3’. For Mojave toxin, published sense and antisense primers were used for both acidic and basic subunits [50]. Twenty-three μL of PCR SuperMix High Fidelity polymerase was used with 1–2 μL of cDNA template and 0.5 μL of each primer (sense and antisense). PCR was performed with seven touchdown cycles of 94°C for 25 seconds, 52°C for 30 seconds, and 68°C for two minutes. Thirty additional cycles followed with 94°C for 25 seconds, 48°C for 30 seconds, and 68°C for two minutes with a final 68°C extension for five minutes. The amplified products were observed on a 1% agarose gel, and bands of the estimated transcript sizes (based on previous published transcripts) were excised and then purified using the Wizard SV gel and PCR clean-up system.

**Table 1:** List of primers used for amplification of transcripts within specific venom protein families.

Venom Protein	Primer sequence	Reference
Rattlesnake phospholipase A_2_	5’-GTCTGGATTCRGGAGGATGAGG-3’	Current study
Elapid phospholipase A_2_	5’-CTGYTGBTGANBKTG-3’	Current study
Rattlesnake metalloproteinase (PII and PIII)	5’-AATCYAGSCTCCAAAATGATC-3’	Current study
Rear-fanged snake metalloproteinase	5’-ATGATCCAAGYTCTCTTGRTWACTATATRCTTAG-3’	Current study
Rattlesnake serine protease	5’-ATGGTGCTGATCAGAGTGCTAGCAAACCTTCT-3’	Current study
Rattlesnake C-type lectin	5’-ATGKGGCRATTSAYC-3’	Current study
Mojave toxin subunit A	Sense: 5’-GGTATTTCGTACTACAGCTCTTACGGA-3’	Wooldridge et al., 2001
	Antisense: 5’-TGATTCCCCCTGGCAATT-3’	
Mojave toxin subunit B	Sense: 5’-AACGCTATTCCCTTCTATGCCTTTTAC-3’	Wooldridge et al., 2001
	Antisense: 5’-CCTGTCGCACTCACAAATCTGTTCC-3’	

R = purine (A or G)

Y = pyrimidine (C or T)

B = not A (C, G, or T)

K = keto (G or T)

S = strong (C or G)

W = weak (A or T)

N = any base (A, C, G, or T).

### Cloning and Sequencing of Venom cDNA

Amplified and purified cDNA was ligated into the pGEM-T Easy Vector System and transformed into *Escherichia coli* DH5 α competent cells following the manufacture’s recommended protocol. Transformed *E*. *coli* were grown on nutrient rich agar plates overnight at 37°C with ampicillin, IPTG and β-galactosidase for white/blue colony selection. Recombinant plasmids were selected from agar plates, and *E*. *coli* colonies picked for viper PLA_2_ sequences were as follows: three colonies were picked for *Crotalus cerastes*, three for *S*. *m*. *barbouri*, four for *C*. *m*. *nigrescens*, six for *C*. *o*. *cerberus*, six for *C*. *basiliscus*, eight for *C*. *pricei*, twelve for *C*. *o*. *concolor*, and fifteen were picked for *C*. *s*. *tzabcan*. In addition to the PLA_2_ sequences, eight colonies for serine proteases, ten colonies for C-type lectins, and eighteen colonies for metalloproteinases were chosen from *C*. *s*. *tzabcan* venom to obtain transcripts for all major venom protein families. For rear-fanged colubrid snake 3FTxs, the following number of *E*. *coli* colonies were selected: three were picked for *T*. *b*. *lambda*, three for *A*. *prasina*, six for *O*. *fulgidus*, four for *B*. *nigriceps*, ten for *B*. *cynodon*, twenty for *B*. *dendrophila*, and nineteen were picked for *B*. *irregularis*. A sampling of 3FTxs from *Boiga sp*. was chosen because of the three currently identified prey-specific 3FTxs, two have been found in *Boiga* species [8, 44]. Three-finger toxin sequences from rear-fanged snakes, especially those from *Boiga sp*. and *O*. *fulgidus*, can provide insight into the evolution of 3FTx prey-specific binding affinities. Three colonies were also picked for metalloproteinase transcripts in *A*. *portoricensis* venom. For the elapid snake *P*. *porphyriacus*, five colonies from amplified PLA_2_s were picked. The numbers of colonies picked varied depending on the number of expected isoforms within each snake venom protein family and also because some primers were still being evaluated for specificity (only a few colonies were selected in these cases). Each *E*. *coli* colony was placed into 2 mL LB broth with 1 μL/mL ampicillin, and shaken overnight at 37°C. Plasmid copies for each *E*. *coli* colony were than purified using the Quick Clean 5M Miniprep Kit and were sequenced at the DNASU facility (Arizona State University, AZ, U.S.A) using Big Dye V3.1 chemistry with samples processed on an Applied Biosystems 3730XL Sequence Analysis Instrument.

### Sequence Analysis

Sequences were viewed with 4Peaks software (http://nucleobytes.com/index.php/4peaks) and base pairs with acceptable quality scores (Phred score >20) were retained for analysis. Redundant sequences were removed. Sequences were identified with BLASTx (Basic Local Alignment Search Tool) on the NCBI server, limiting the search to “Serpentes (taxid: 8570)” proteins. Protein identities were considered significant if they fell below an e-value threshold of e^-4^ and shared sequence similarity to other known snake venom proteins. Sequences were translated to their corresponding amino acid sequence and trimmed in MEGA v6.06 [48], then aligned with MUSCLE [49] and manually checked. Sequence alignment figures were generated using BoxShade 3.3.1 (http://mobyle.pasteur.fr/cgi-bin/portal.py?#forms::boxshade). All full-length CDS sequences, including all rattlesnake PLA_2_s, *C*. *s*. *tzabcan* serine proteases, *C*. *s*. *tzabcan* C-type lectins, and rear-fanged snake three-finger toxin sequences, were submitted to GenBank (accessions KU666900-KU666937).

Phylogenetic analysis was completed with MrBayes v3.2.4 [51] using models selected by PartitionFinder v1.1 [52]. PartitionFinder v.1.1 models selected were favored using Akaike Information Criterion. These datasets were then run in duplicate using MrBayes v3.2.4 with the default of three heated and one cold chain for 1x10^7^ generations, sampling every 1,000 generations, and with the first 10% discarded as burn-in. Tracer v1.6 (http://tree.bio.ed.ac.uk/software/tracer/) was used to check for run convergences. Consensus tree figures were prepared with FigTree v1.4.0 (http://tree.bio.ed.ac.uk/software/figtree/).

## Results

### Venom RNA Isolation

Venom RNA concentrations were determined using both a Nanodrop 2000 (Thermo Fisher Scientific, NY, U.S.A) and a Qubit 2.0 Fluorometer (Life Technologies, CA, U.S.A) with a high sensitivity RNA assay kit. Various RNA isolation kits and reagents were used to determine which method produced the greatest RNA yields and amplification success (Table 2). The TRIzol RNA isolation protocol described in the methods was found to produce the most consistent results when isolating venom RNA and amplifying transcripts; however, the Direct-zol RNA kit (Zymo Research, CA, U.S.A) and *mir*Vana miRNA isolation kit (Life Technologies, CA, U.S.A) were also successful. Dynabeads from Life Technologies, CA, U.S.A were tried using a previously published technique for isolating extracellular mRNA within venom [29, 30], as well as a FastTrack MAG mRNA isolation kit (Life Technologies, CA, U.S.A) and a RNeasy mini kit (QIAGEN, CA, U.S.A), but cDNA amplification did not produce visible PCR products (Table 2).

**Table 2:** RNA and mRNA isolation protocols used to obtain extracellular RNA within venom, and the resulting yields and cDNA amplification success.

RNA or mRNA isolation procedure	Total RNA or mRNA yields (Nanodrop)		Total RNA or mRNA yields (Qubit)	Successful cDNA amplification
TRIzol	1.10–13.60 μg (RNA)	< 20 ng/μl (RNA)		+
*mir*Vana miRNA isolation kit	0.33–1 μg (RNA)	< 20 ng/μl (RNA)		+
Direct-zol RNA kit	0.08–0.13 μg (RNA)	nd		+
RNeasy mini kit	0.08–0.30 μg (RNA)	nd		−
Dynabeads	0.20–1.19 μg (mRNA)	nd		−
FastTrack MAG mRNA isolation kit	0.01–0.03 μg (mRNA)	nd		−

nd = not determined

+ = successful amplification

- = unsuccessful.

**Fig 1:**
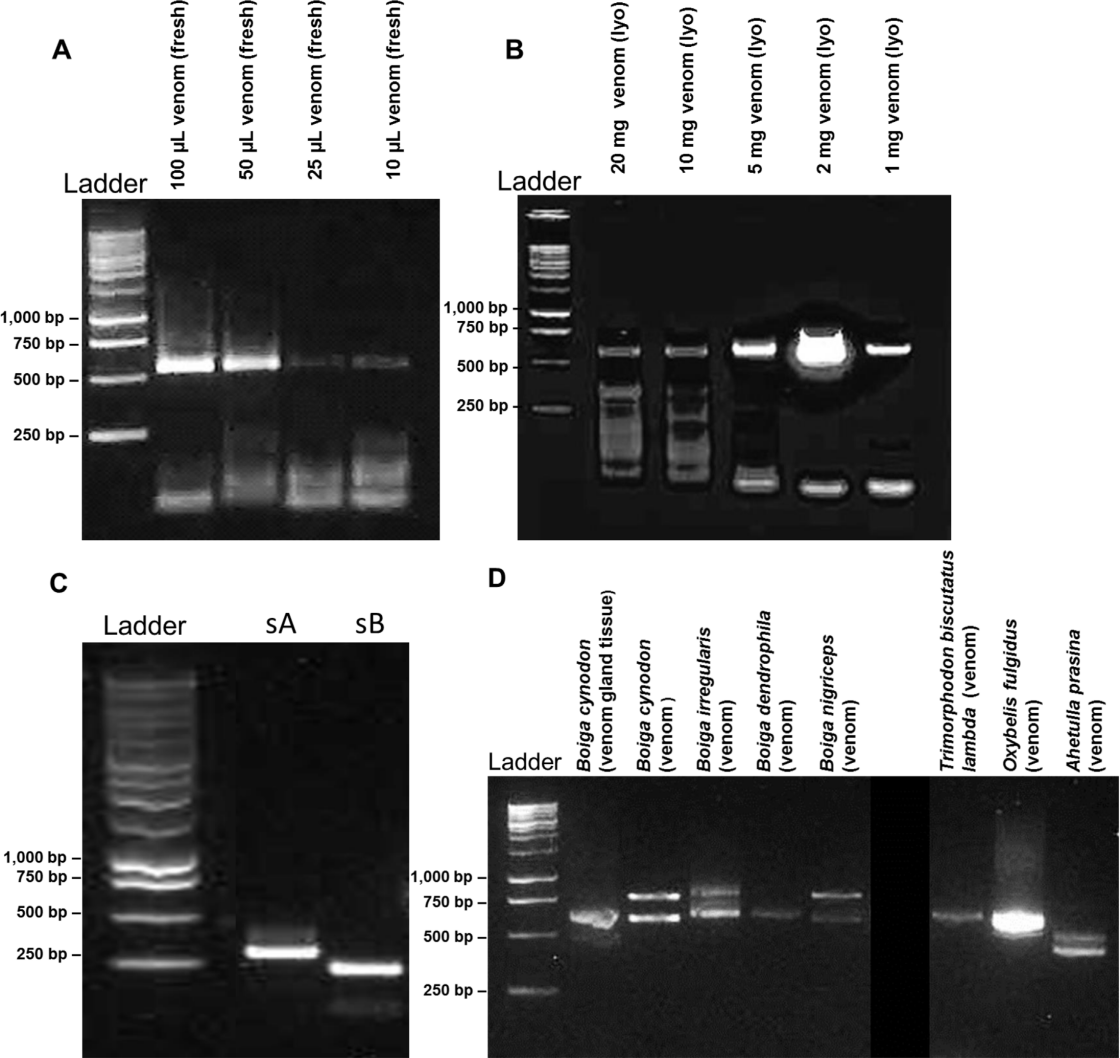
Agarose gel electrophoresis showing cDNA transcripts amplified from mRNA in venomous snake venoms. Various amounts of fresh venom (A) and lyophilized venom (B) from *Crotalus simus tzabcan* were used to determine optimal amplification conditions for phospholipase A_2_ transcripts. Regions of phospholipase A_2_ Mojave toxin subunit A (sA) and subunit B (sB) cDNAs were amplified to demonstrate transcript detectability in *Crotalus scutulatus scutulatus* venom (C). Three-finger toxin cDNA sequences were also amplified from mRNA derived from venoms of rear-fanged snakes (D).

The TRIzol reagent Nanodrop readings for RNA yields of different rattlesnake venoms varied from 69 ng/μl (1.1 μg of total RNA isolated from 2 mg of lyophilized rattlesnake venom) to 683.3 ng/μl (10.9 μg of total RNA isolated from 100 μl of fresh rattlesnake venom) (Table 2). Rear-fanged snake venoms consistently showed slightly higher yields (10.3 μg– 13.6 μg of total RNA) as determined by Nanodrop; however, this appeared to be mostly due to the 260 nm readings of contaminating DNA from saliva during venom collection, and all RNA isolated from rear-fanged snakes required a DNase I digestion before PCR to prevent nonspecific amplification. When fresh venom was used, 100 μl of *C*. *s*. *tzabcan* venom yielded a total RNA amount (Nanodrop) of 6.1 μg, 50 μl yielded 8 μg, and 25 μl yielded 10.4 μg. However, when the same volume amounts were used for cDNA synthesis and amplification, successful amplification of PLA_2_ transcripts tended to decrease with decreasing venom input (Fig 1A). When different lyophilized venom amounts were used, 20 mg yielded 6.1 μg, 10 mg yielded 5.2 μg, 5 mg yielded 6.5 μg, 2 mg yielded 5.3 μg, and 1 mg yielded 8.4 μg of total RNA (Nanodrop). As seen with the total RNA amounts from fresh venom, the Nanodrop amount of total RNA from lyophilized venom did not demonstrate a clear relationship to PLA_2_ transcript amplification success, and 2 mg produced the highest concentration of PLA_2_ amplicons (Fig 1B). All Nanodrop readings showed low 260/280 and 260/230 ratios, indicating low purity. The typical 260/280 ratio observed was 1.5 and 1.6 for 260/230. Qubit results revealed values < 20 ng/μl, below instrument detection, for all measured samples (Table 2).

It was possible to isolate RNA from both fresh venom and from lyophilized venom that had been stored at -20°C (including after 20 years of storage), as well as from venom desiccated in the field and venom purchased from a commercial venom supply source. Both front-fanged and rear-fanged venomous snakes were found to have extracellular RNA within their venoms; this is the first report of mRNA in the venom of rear-fanged venomous snakes, and RNA was isolated from both freshly collected and lyophilized rear-fanged snake venoms.

### Venom Protein cDNA Sequences within Venom

As proof of concept, cDNA amplicons from *C*. *s*. *scutulatus* venom, obtained using published primer sequences [49], were used to confirm presence of the two Mojave toxin subunits (acidic and basic chains; Fig 1C). These basic and acidic subunits were both sequenced and found to be 100% identical to the published sequences for *C*. *s*. *scutulatus* (PA2A_CROSS and PA2Ba_CROSS) [53, 54], demonstrating that cDNAs of mRNA within venom can be used to detect the presence of specific expressed venom protein transcripts. In this case, the presence and abundance of crotoxin/Mojave toxin-like acidic and basic subunits is strongly indicative of neurotoxic envenomation symptoms characteristic of human envenomations by these rattlesnakes.

**Fig 2:**
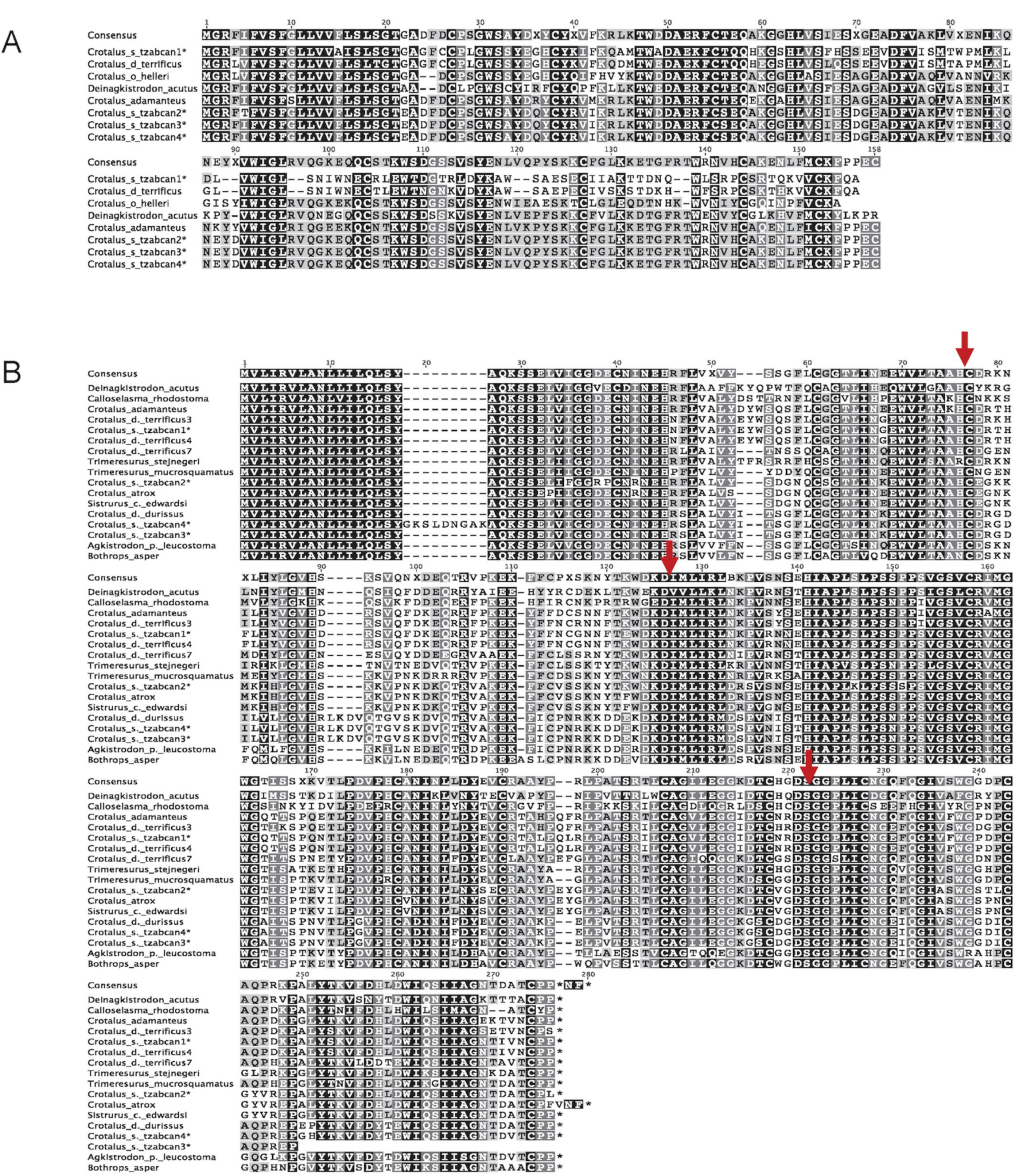
**Aligned Middle American Rattlesnake (*Crotalus simus tzabcan*) C-type lectins (A) and serine proteases (B).** A) Four unique venom-based C-type lectin transcripts (asterisks) were identified for *C*. *s*. *tzabcan* and aligned to other crotaline species. Identical nucleotide sequences are shaded and corresponding GenBank accession numbers are as follows: Crotalus_adamanteus (AEJ31974.1), Deinagkistrodon_acutus (AAM22790.1), Crotalus_d_terrificus (Q719L8.1), and Crotalus_o_helleri (AEU60004.1). B) Venom-based serine proteases cDNA sequences (asterisks) were also obtained from *C*. *s*. *tzabcan* and were aligned with toxins from several other species; identical nucleotide sequences are shaded, and the catalytic triad composed of Ser195, Asp102, and His57 associated with thrombin-like activity in snake venom serine proteases are identified (arrowheads). Isoform 3 from *C*. *s*. *tzabcan* is a partial sequence. GenBank accession numbers are as follows: Agkistrodon_p_leucostoma (HQ270466.1), Bothrops_asper (DQ247724.1), Crotalus_d_terrificus7 (EU360954.1), Crotalus_d_terrificus4 (EU360952.1), Crotalus_d_terrificus3 (EU360951.1), Crotalus_d_durissus (DQ164401.1), Sistrurus_c_edwardsi (DQ464239.1), Trimeresurus_mucrosquamatus (X83225.1), Crotalus_adamanteus (HQ414118.1), Calloselasma_rhodostoma (L07308.1), Deinagkistrodon_acutus (AY861382.1), Trimeresurus_stejnegeri (AF545575.1), and Crotalus_atrox (AF227153.1).

3’RACE with sense primers designed from conserved sequences of the signal peptide or the 5’UTR (untranslated region) of transcripts (Table 1, S1 Fig) were used to amplify cDNAs for a diversity of PLA_2_s, metalloproteinases, serine proteases, C-type lectins, and 3FTxs from viperid, elapid and rear-fanged snake venoms. This is the first time that cDNA derived from venom transcripts has been used to obtain unknown sequences for such a diversity of venom protein families. For Middle American Rattlesnake venom (*C*. *s*. *tzabcan)*, full-length cDNA sequences were successfully amplified for the major venom proteins present within this rattlesnake’s venom [55] in spite of limited colony sampling (Table 3). A partial venom gland transcriptome was assembled, focusing on venom protein transcripts that significantly contribute to envenomation symptomology, including metalloproteinases, serine proteases, and C-type lectins. There are likely many more unique C-type lectins, serine proteases, and metalloproteinase transcripts within *C*. *s*. *tzabcan* venom, but the intent here was to demonstrate the presence of diverse, intact venom protein transcripts (Fig 2). Greater diversity of C-type lectins has been identified within other rattlesnake venom gland transcriptome assemblies using next generation sequencing [23, 25, 27], but the vast majority of these C-type lectins transcripts were found to be present in very low abundance.

**Table 3:** Venom protein transcripts amplified from the venom of the Middle American Rattlesnake (*Crotalus simus tzabcan*) for the four dominant protein families present.

Venom Protein Family	# Colony Picks	# Unique Isoforms
Metalloproteinase (PII and PIII)	18	4
Phospholipase A_2_	15	4
C-type Lectin	10	4
Serine Protease	8	4

The diversity of PLA_2_ isoforms appeared to vary for each rattlesnake species (Table 4). For *C*. *pricei*, only one unique PLA_2_ sequence was discovered in eight selected clones, while *C*. *m*. *nigrescens* had three unique sequences found in the selection of only four clones. There was no clear positive trend between the number of colonies sequenced and the number of unique sequences (Pearson’s correlation test; df = 6, r = 0.6684, p = 0.0699); instead, isoform diversity appeared to be dependent on the species (and likely on the abundance of the most prominent isoform). The number of clones sampled in this study was relatively low, and an increase in the number of clones sequenced should increase the chance of observing less abundant isoforms and determining the total number of isoforms present in each venom [19, 56].

**Table 4:** Relationship between the number of colony picks and the number of observed unique phospholipase A_2_ isoforms.

Species	# Colony Picks	# Unique PLA_2_ Isoforms
*Crotalus simus tzabcan*	15	4
*Crotalus oreganus concolor*	12	3
*Crotalus pricei pricei*	8	1
*Crotalus oreganus cerberus*	6	2
*Crotalus basiliscus*	6	3
*Crotalus molossus nigrescens*	4	3
*Crotalus cerastes cercobombus*	3	1
*Sistrurus miliarius barbouri*	3	1
*Pseudechis porphyriacus*	5	2

**Fig 3:**
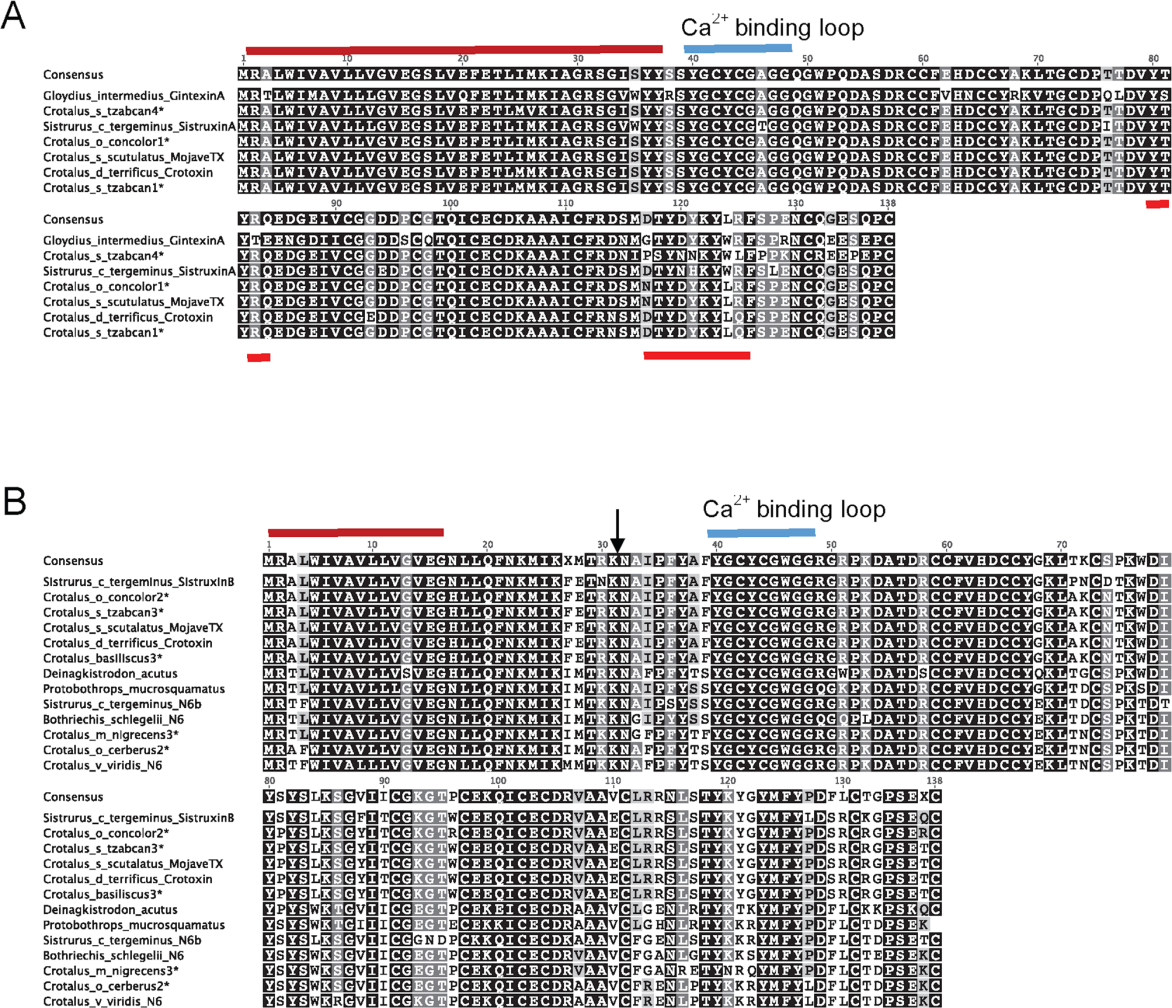
**Aligned Group IIA phospholipase A_2_ acidic (A) and basic (B) subunit isoforms.** The neurotoxic, heterodimeric PLA_2_ complex of crotoxin/Mojave toxin homologs consists of both an acidic A subunit and basic B subunit. A) Sequence alignments of the acidic (A) subunit of crotoxin or Mojave toxin homologs with identical residues shaded; the conserved signal peptide region is indicated by the red bar, and the Ca^2+^ binding loop is indicated by the blue bar. Regions of the A subunit which are post-translationally cleaved in the mature protein are indicated by red brackets below sequences. Sequences similar to crotoxin/Mojave toxin acidic subunit A derived from from venom (asterisks) were discovered in *Crotalus oreganus concolor* and *C*. *simus tzabcan* venoms. GenBank accession numbers of known toxins are as follows: Crotalus_s_scutulatus_MojaveTX (U01026.1), Crotalus_d_terrificus_CrotoxinA (X12606.1), Sistrurus_c_tergeminus_SistruxinA (Q6EAN6.1), and Gloydius_intermedius_GintexinA (AID56658.1). B) Sequence alignments of the basic (B) subunit of crotoxin/Mojave toxin homologs with identical residues shaded; the conserved signal peptide region is indicated by the red bar, and the Ca^2+^ binding loop is indicated by the blue bar. The asparagine-6 (N6) associated with neurotoxic PLA_2_ functionality is indicated by arrowheads. Sequences similar to the crotoxin or Mojave toxin basic B subunits derived from venom (asterisks) were discovered in *Crotalus oreganus concolor*, *C*. *simus tzabcan*, and *C*. *basiliscus* venoms, with N6 sequences also found in *C*. *m*. *nigrescens* and *C*. *o*. *cerberus* venoms. GenBank accession numbers of known toxins are as follows: Crotalus_s_scutulatus_MojaveTX (U01027.1), Crotalus_d_terrificus_CrotoxinB (X12603.1), Crotalus_v_viridis_N6 (AF403138.1), Sistrurus_c_tergeminus_N6 (AY355169.1), Sistrurus_c_tergeminus_SistruxinB (Q6EER2.1), Bothriechis_schlegelii_N6 (AY355168.1), Protobothrops_mucrosquamatus (AF408409.1), and Deinagkistrodon_acutus (X77649.1).

Of the fifteen PLA_2_ clones selected from *C*. *s*. *tzabcan*, two unique sequences were similar to sequences from crotoxin or Mojave toxin-like acidic A chain (Fig 3). One sequence (C_s_tzabcan1) was the most abundant, with six identical clones, and it was 99% identical in amino acid sequence to crotoxin acidic A chain (PAIA_CRODU) from the South American Rattlesnake (*Crotalus durissus terrificus*), while the second sequence (C_s_tzabcan4) had only one clone and was 88% identical to the Mojave toxin acidic (A) chain (PA2A_CROSS) from the Mojave Rattlesnake (*C*. *s*. *scutulatus*). This less abundant acidic chain sequence revealed that isoform variation within the acidic (A) chains of the toxin also exists for *C*. *s*. *tzabcan*.

A crotoxin-like basic (B) chain was also sequenced from *C*. *s*. *tzabcan* venom, with three clones that were 100% identical in amino acid sequence to crotoxin subunit CBc from *C*. *d*. *terrificus* (PA2BC_CRODU), providing molecular evidence that *C*. *s*. *tzabcan* from this study has an abundance of available PLA_2_ transcripts to form a neurotoxic complex similar to that of *C*. *d*. *terrificus* (Fig 3). The PLA_2_ cDNAs obtained from *C*. *basiliscus* also had multiple clones containing crotoxin-like basic B chain sequences (C_basiliscus3), which were 100% identical in amino acid sequence to crotoxin basic subunit CBc of *C*. *d*. *terrificus* (PA2BC_CRODU) (Fig 3B). However, crotoxin-like acidic subunit sequences were not discovered, either due to a lack of sufficient sampling or absence from this venom sample. Crotoxin-like protein complexes have been previously observed in *C*. *basiliscus* venom [57].

Mojave toxin-like PLA_2_ sequences were obtained from *C*. *o*. *concolor* venom (Fig 3), which has been previously recognized as containing a neurotoxic PLA_2_ complex (concolor toxin) similar to Mojave toxin, identified from Ouchterlony immunodiffusion, immunoelectrophoresis, ELISA, and Western blot analyses [58, 59]; however, the full sequence has not been published. Concolor toxin acidic (A) chain was found to share 100% sequence identity with Mojave toxin acidic (A) subunit (PA2A_CROSS) from *C*. *s*. *scutulatus;* however, the concolor basic (B) subunit was found to be more similar to crotoxin subunit CBc (PA2BC_CRODU) from *C*. *d*. *terrificus*, sharing 99% amino acid sequence identify with this crotoxin basic (B) chain.

Interestingly, *C*. *o*. *cerberus* and *C*. *m*. *nigrescens* venoms also had PLA_2_ sequences that contained the asparagine-6 (N6) substitutions associated with myotoxic/neurotoxic activity, also a feature of the basic (B) subunits of crotoxin and Mojave toxin (Fig 3). All other PLA_2_ sequences were similar in sequence to acidic PLA_2_s that show edema-inducing activity and myotoxicity, corresponding to the envenomation symptomology seen in bites from these species. The *S*. *m*. *barbouri* PLA_2_ sequence reported here was found to be 100% identical to the amino acid sequence of a previously reported PLA_2_ from *S*. *m*. *barbouri* venom (ABY77929.1 [60]).

**Fig 4:**
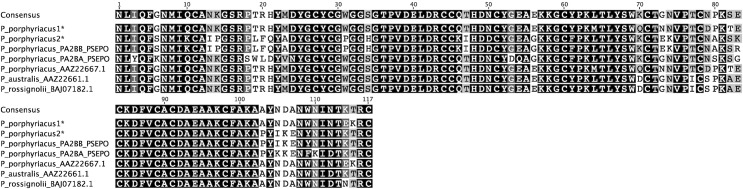
Aligned *Pseudechis* Group IA phospholipase A_2_ (PLA_2_) mature protein sequences. Amplified PLA_2_ sequences from *Pseudechis porphyriacus* venom (asterisks) were aligned to known PLA_2_ sequences from the same species and known PLA_2_ sequences from two other *Pseudechis* species. Sequences from *P*. *porphyriacus* venom showed 99% and 100% identity to *P*. *porphyriacus* mature PLA_2_ protein sequences that had been obtained from venom gland tissue or via Edman degradation.

There were only two unique PLA_2_ transcripts identified in five colonies selected from an elapid (*P*. *porphyriacus*) venom (Table 3). However, of the identified PLA_2_ sequences, 4/5 clones were 100% identical in mature protein sequence (P. porphyriacus1) to a previously identified PLA-1 precursor (AAZ22667.1) from *P*. *porphyriacus* venom gland [61], and the other unique isoform (P. porphyriacus2) was 99% identical to PLA_2_ pseudexin B chain (PA2BB_PSEPO), also from *P*. *porphyriacus* [62]. Again, sequences determined from venom-derived mRNAs are identical to previously reported venom protein sequences (Fig 4), validating this method.

**Fig 5:**
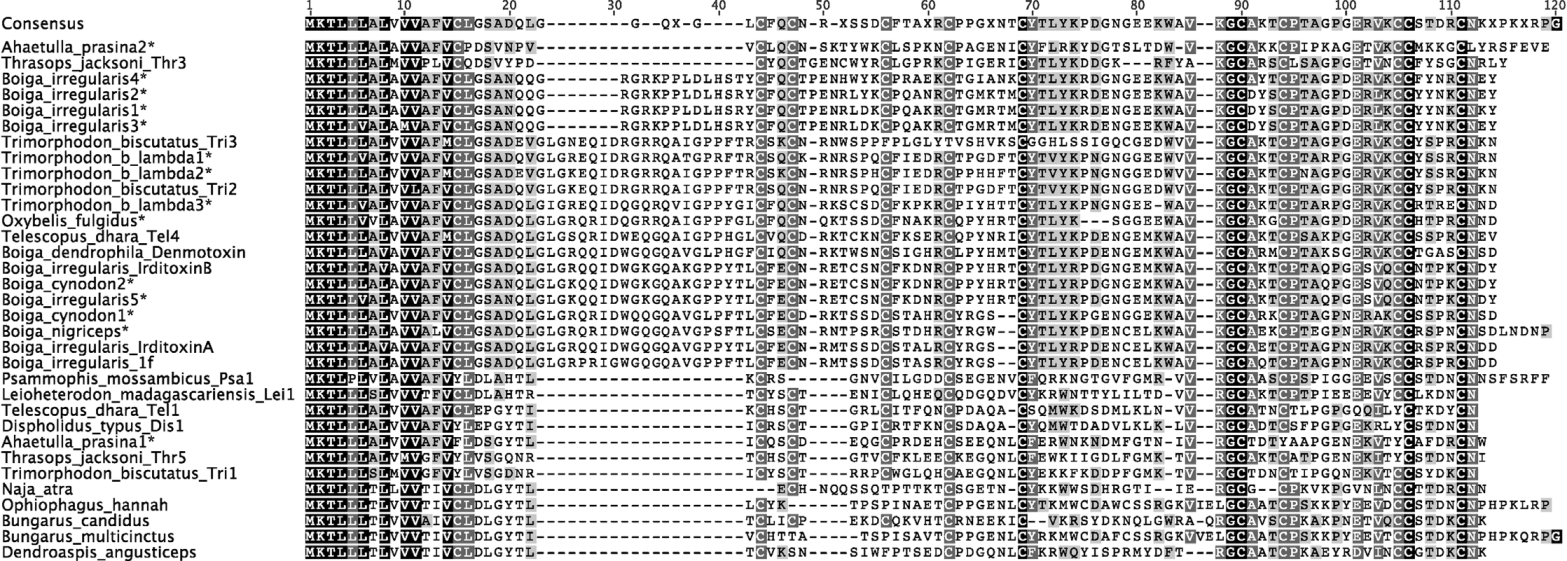
Aligned sequences of non-conventional three-finger toxins (3FTxs) from venoms of rear-fanged and elapid snakes. Venom-derived 3FTx sequences (asterisks) obtained from *Boiga irregularis*, *B*. *dendrophila*, *B*. *nigriceps*, *B*. *cynodon*, *Oxybelis fulgidus*, *Ahaetulla prasina*, and *Trimorphodon biscutatus lambda* were aligned with various other rear-fanged and Elapidae species; identical nucleotide sequences are shaded. GenBank accession numbers are as follows: Trimorphodon_biscutatus_Tri3 (EU029678.1), Trimorphodon_biscutatus_Tri2 (EU029677.1), Telescopus_dhara_Tel4 (EU029686.1), Boiga_dendrophila_denmo (DQ366293.1), Boiga_irregularis_irditoxinB (DQ304539.1), Boiga_irregularis_irditoxinA (DQ304538.1), Boiga_irregularis_1f (GBSH01000015.1), Thrasops_jacksoni_Thr3 (EU029685.1), Dispholidus_typus_Dis1 (EU029674.1), Telescopus_dhara_Tel1 (EU029675.1), Thrasops_jacksoni_Thr5 (EU036635.1), Trimorphodon_biscutatus_Tri1 (EU029675.1), Naja_atra (AF031472.1), Bungarus_multicinctus (AF056400.1), Ophiophagus_hannah (FJ952515.1), Psammophis_mossambicus_Psa1 (EU029669.1), Leioheterodon_madagascariensis (EU029676.1), Bungarus_candidus (AY057878.1), and Dendroaspis_angusticeps (AF241871.1).

Full-length venom protein transcripts were also identified from rear-fanged snake venoms. Thirty full-length 3FTx sequences were obtained using a degenerate sense primer designed from multiple sequence alignments with published non-conventional 3FTx sequences (Fig 1D, S1B Fig). Three-finger toxin transcripts were found in the venoms of *T*. *b*. *lambda*, *A*. *prasina*, *O*. *fulgidus*, *B*. *nigriceps*, *B*. *cynodon*, *B*. *dendrophila*, and *B*. *irregularis* (Fig 5).

**Fig 6:**
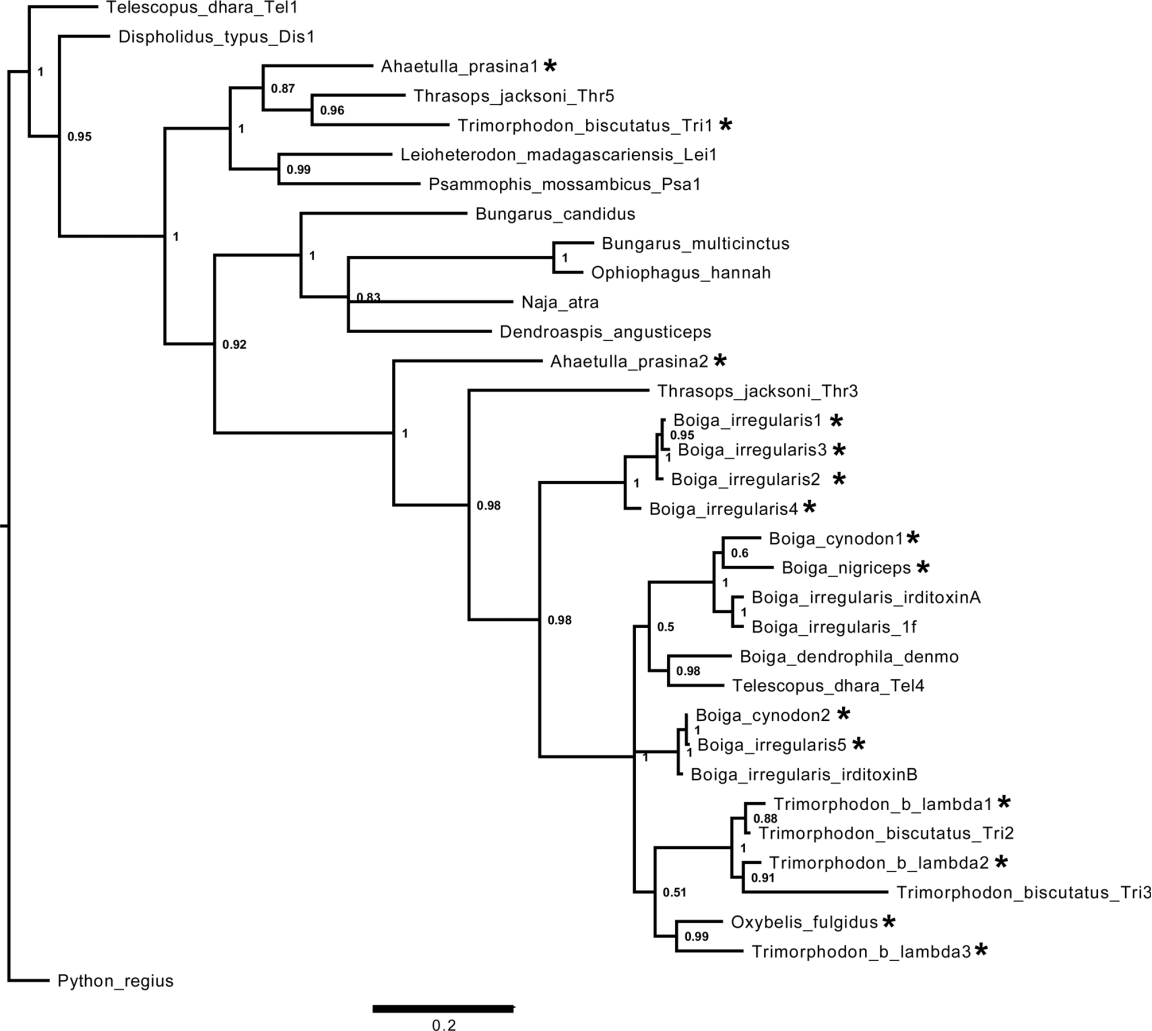
Bayesian sequence similarity tree depicting non-conventional three-finger toxin (3FTx) relationships. 3FTx cDNA sequences derived from venom (asterisks) were obtained from *Boiga irregularis*, *B*. *dendrophila*, *B*. *nigriceps*, *B*. *cynodon*, *Oxybelis fulgidus*, *Ahaetulla prasina* and *Trimorphodon biscutatus lambda*, and sequence relationships with various known rear-fanged and Elapidae 3FTxs are shown. Posterior probabilities are shown for each node, and GenBank accession numbers correspond to sequences in Fig 6.

Although none of the three 3FTx sequences from *T*. *b*. *lambda* venom were 100% identical to previously published 3FTx sequences from this species [63], they did cluster with the previous *T*. *b*. *lambda* sequences within a well-supported clade also containing other 3FTx sequences from New World rear-fanged venomous snakes (Fig 6). A greater diversity of 3FTxs within *T*. *b*. *lambda* venom is possible considering that only three clones were picked for this study and all were unique 3FTx sequences; differences observed could also be due to locality-specific transcript variation. Two unique 3FTx sequences were found in *A*. *prasina* venom and only one unique 3FTx sequence in *O*. *fulgidus*. The sequence from *O*. *fulgidus* venom was not identical to the previously characterized fulgimotoxin, which was based on N-terminal Edman degradation sequencing [45]; however, it did show 95% amino acid sequence identity.

Only two unique 3FTx sequences were found in the venom of *B*. *cynodon* (from ten selected colonies) and one in *B*. *nigriceps* venom (from four selected colonies). Many of the clones sequenced from rear-fanged snake venoms were of poor quality and were culled, so more sequences are likely present. Fifteen unique 3FTx sequences were revealed in *B*. *dendrophila* venom, but the majority were missing complete signal peptide sequences and therefore were omitted from further analysis because it is unknown if these transcripts produce proteins that are secreted in the venom gland and are active components of *B*. *dendrophila* venom. Six unique 3FTx sequences were found in *B*. *irregularis* venom, but none were 100% identical to either irditoxin subunits [8], although one sequence was 96% identical to irditoxin subunit B. One of the 3FTx *B*. *cynodon* clones was 97% identical (amino acid sequence) to irditoxin subunit B and another sequence had 83% amino acid sequence identity with irditoxin subunit A; these toxins also clustered together with irditoxin in the 3FTx phylogenetic tree (Fig 6). These results suggest that *B*. *cynodon* venom likely contains a prey-specific heterodimeric 3FTx complex similar to irditoxin.

**Fig 7:**
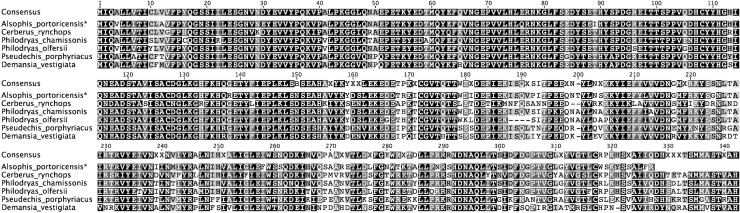
*Alsophis portoricensis* venom PIII metalloproteinase sequence aligned with amino acid sequences from rear-fanged and elapid snake species. Identical residues are shaded, demonstrating PIII metalloproteinase sequence conservation in these diverse species. Only a partial sequence of the complete transcript from *A*. *portoricensis* (asterisks) was used for the alignment. Genbank accession numbers are as follows: Philodryas_chamissonis (AJB84503.1), Philodryas_olfersii (ACS74987.1), Cerberus_rynchops (ADJ51055.1), Pseudechis_porphyriacus (ABQ01133.1), and Demansia_vestigiata (ABK63559.1).

One unique metalloproteinase sequence was amplified, cloned and sequenced from the venom of the Puerto Rican Racer (*Alsophis portoricensis*). Although this sequence was not similar to alsophinase, a previously characterized metalloproteinase in *A*. *portoricensis* venom [43], it was similar in sequence to other rear-fanged and elapid P-III metalloproteinase cDNA sequences (Fig 7). The complete metalloproteinase sequence was amplified, based on the observed amplified product size, but longer venom protein transcripts (>2,000bp) required multiple sequencing reactions that were not performed for this analysis, and therefore only the partial sequence is shown in the alignment (Fig 7).

### Other cDNA Sequences within Venom

While optimizing primers, other sequences were incidentally amplified from both rattlesnake and rear-fanged snake venom, including complete 60S ribosomal sequences. These sequences, from *C*. *cerastes* and *A*. *portoricensis* venom, were 99% identical to the predicted Burmese Python (*Python bivittatus*) 60S ribosomal protein L7a (XP_007420634.1) and L15 isoform X1 (XP_007421748.1), respectively. There were also 40S ribosomal protein sequences amplified from *C*. *s*. *scutulatus* which showed 100% sequence identity with the 40S ribosomal protein S9-like isoform X1 from *P*. *bivittatus* (XP_007439934.1). Cathelicidin-OH antimicrobial peptides (XP_007442672.1) were identified from *C*. *o*. *cerberus* and *B*. *irregularis* venom. These sequences were observed in both rattlesnake and rear-fanged snake venoms, demonstrating that other complete transcripts, in addition to venom protein transcripts, exist within venoms (S2 Fig).

### Relationship of Sequence Similarities to Empirical and Predictive Protein Activities

**Fig 8:**
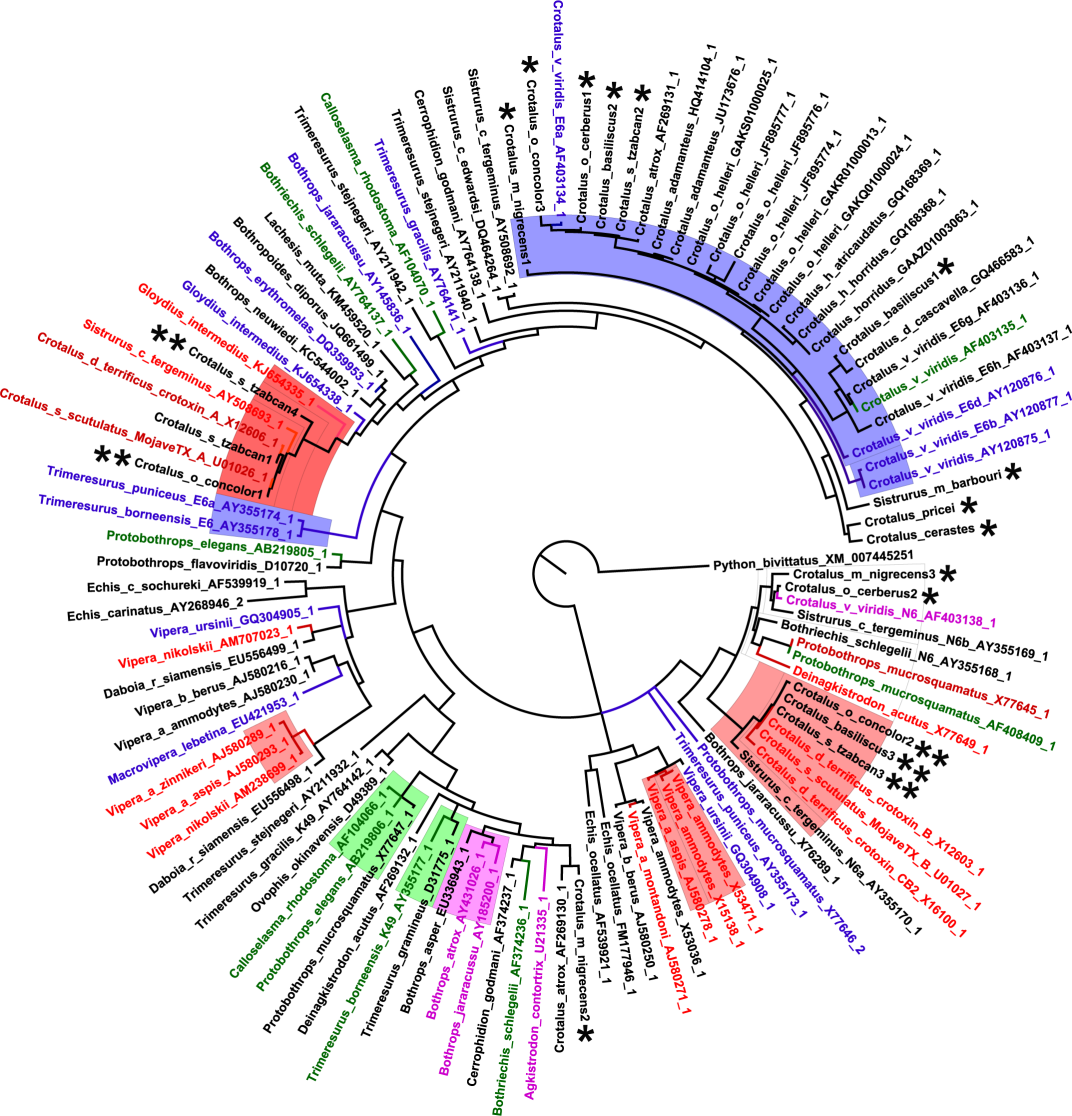
Bayesian sequence similarity tree depicting viper Group II phospholipase A_2_ (PLA_2_) relationships. PLA_2_ cDNA sequences were obtained from *Crotalus simus tzabcan*, *C*. *cerastes*, *C*. *molossus nigrescens*, *C*. *oreganus concolor*, *C*. *oreganus cerberus*, *C*. *pricei*, *C*. *basiliscus*, *and Sistrurus miliarius barbouri* venoms (asterisks), and their relationships with various published Old and New World viperid venom PLA_2_s are shown. Newly sequenced crotoxin homologs are indicated by double asterisks (crotoxin A—left side; crotoxin B–right side). GenBank accession numbers are listed within the tree. Published PLA_2_ sequences are colored corresponding to empirically determined biological activity. Whole clades are highlighted where at least two of the published sequences within the clade have the same experimentally determined activity. Red, neurotoxic; blue, hemolytic; green, edematous; pink, myotoxic.

Sequences that were similar to crotoxin/Mojave toxin acidic (A) subunits in *C*. *o*. *concolor* and *C*. *simus tzabcan* venoms formed one well-supported clade (1.0 posterior probability), and sequences that were similar to crotoxin/Mojave toxin basic (B) subunits discovered in *C*. *o*. *concolor*, *C*. *simus tzabcan*, and *C*. *basiliscus* clustered with other known neurotoxic N6 PLA_2_ homologs (1.0 posterior probability; Fig 8). Other PLA_2_s from *C*. *o*. *concolor*, *C*. *simus tzabcan*, and *C*. *basiliscus* venoms, and also from *C*. *pricei*, *C*. *cerastes*, *C*. *m*. *nigrescens*, *C*. *o*. *cerberus*, and *S*. *m*. *barbouri* venoms, clustered within an acidic hemolytic PLA_2_ clade shared with other rattlesnakes (0.96 posterior probability; Fig 8). It has been experimentally determined that even neurotoxic PLA_2_s can also exhibit anticoagulant activity, and this appears to be a common characteristic of many venom PLA_2_ enzymes.

## Discussion

Concentrations of total extracellular RNA within snake venom were observed to be moderate but variable using Nanodrop. Using a Qubit instrument, RNA concentrations were all below the instrument detection limit (< 20 ng/μl) (Table 2). Qubit readings provide better accuracy because the fluorescent dye is highly selective for RNA and not DNA, which has also been detected in venom [64]. In addition, common contaminants do not affect Qubit readings. The more accurate Qubit readings revealed a much lower concentration of extracellular RNA within venom, lower than Nanodrop concentrations reported in this and in a previous study [30]. However, in spite of low RNA concentrations, venom protein cDNAs could still be amplified successfully from both front and rear-fanged venomous snakes. By using extracellular messenger RNA from venom to obtain full-length venom protein transcripts, this method can be used without the need to sacrifice living animals to obtain venom gland tissue. It was also possible to successfully amplify full-length venom protein transcripts from venom that was fresh, lyophilized or stored at -20°C for 20+ years, desiccated over silica gel in the field, or obtained from a commercial venom supplier. This represents a significant advance over previous attempts to amplify venom-derived mRNAs, which typically produced only partial sequence transcripts [29, 30].

Venom protein genes experience an accelerated rate of nucleotide substitution [65, 66], making it difficult to design sense and antisense pairs of primers to amplify unknown venom proteins sequences, which is why complete venom gland transcriptomes from gland tissue are usually assembled. However, venom proteins demonstrate high conservation of nucleotide signal peptide sequence and/or 5’UTRs (Fig 2). By designing degenerate sense primers from these conserved nucleotide sequences and performing 3’RACE, the successful amplification of a diversity of transcript sequences for the major venom protein families (metalloproteinases, serine proteases, C-type lectins, phospholipase A_2_s, and three-finger toxins) responsible for clinically significant snakebite were obtained from venom. This approach also allowed determination of unique, currently unknown full-length toxin sequences for many front-fanged and rear-fanged species in all of the major clades of venomous snakes (Viperidae, Elapidae, Colubridae). This use of degenerate primers to amplify unknown full-length venom protein sequences within a superfamily from snake venom can be employed to screen sequences within each species for toxins of interest, to examine novel mutations within a venom protein superfamily, or to provide an inexpensive method to obtain complete amino acid sequence for a protein under investigation.

Venom gland transcriptomes generated from next-generation sequencing (Roche 454 or Illumina) provide more comprehensive transcriptome profiles and identify the complete repertoire of transcripts within each venom protein superfamily [23, 25, 27]. An abundance of unique 3FTx transcripts identified in rear-fanged snake venom gland transcriptomes generated by next-generation sequencing has been reported, with over fifty 3FTxs transcripts in the case of *Boiga irregularis* [28]. The number of unique venom protein PLA_2_ sequences discovered in viper venom gland transcriptomes completed with next-generation sequencing ranges from 4–9 [23, 25, 27]; therefore, the number of unique sequences obtained in this study was by no means a comprehensive evaluation of all transcripts within these protein superfamilies. However, using established procedures that are readily accessible to many researchers, such as 3’RACE and the selection/sequencing of *E*. *coli* clones, it was possible to identify the major transcripts present for each venom protein superfamily explored in this study. The approach used here allows for researchers interested in a single venom protein superfamily to obtain selectively all highly abundant transcripts for that protein superfamily. This approach is cost-effective and does not require the computing resources/bioinformatics needed for next generation sequencing transcriptome assemblies. Because venom protein cDNA sequences are obtained from venoms, this method also allows for the assembly of a genotype-phenotype map, using only venom as source material.

Phospholipase A_2_ enzymes and 3FTxs were chosen as the main focus of this study because they constitute very large venom protein superfamilies that exhibit a diversity of activities, including neurotoxic, myotoxic, cardiotoxic, anticoagulant and hemolytic activities [2, 67, 68]. These venom proteins are ideal for structure/function studies as well as protein engineering studies, because a variety of activities and functional sites are possible using the same conserved protein structural scaffold. Also, 3FTxs and PLA_2_s are venom proteins that are observed in abundance in snake venoms, and both are toxins that contribute significantly to serious snake envenomation symptomology. Presence of crotoxin or Mojave toxin PLA_2_ heterodimeric complexes result in phenotypically neurotoxic venoms, and the absence or presence of these complexes result in distinctive venom types that have been labeled type I and type II. Type I venoms have higher metalloproteinase activity and lower toxicity, and type II venoms have low metalloproteinase activity and high toxicity/neurotoxicity [69].

There can be variation in the occurrence of crotoxin/Mojave toxin complexes within a species, as is seen among different populations of *C*. *horridus*, *C*. *scutulatus* and *C*. *simus* throughout their range [55, 70, 71]. This study shows that it is possible to detect the acidic and basic subunit transcripts of these neurotoxic PLA_2_ complexes within venom. In the case of *C*. *s*. *tzabcan* utilized in this study, several isoforms of acidic and basic crotoxin-like subunits were observed. The neurotoxicity of *C*. *s*. *tzabcan* venom varies with snake locality [58]; because the specific locality of the *C*. *s*. *tzabcan* used in this study was unknown, sequencing venom protein transcripts present within venom was a successful approach to evaluating venom phenotype.

This technique can also be used to analyze the amino acid sequences of toxins in unexplored venoms, and this study is the first to report the complete sequence for both subunits of concolor toxin from venom of *C*. *o*. *concolor*. Although it has been known for some time that this neurotoxic complex is present in *C*. *o*. *concolor* venom [58, 59], the presence of acidic and basic crotoxin/Mojave toxin homologs confirmed that a PLA_2_-based neurotoxin was present in this type II venom. The viper PLA_2_ Bayesian sequence similarity tree revealed some distinctive clusters that corresponded with experimentally characterized PLA_2_ protein activities. For example, analysis of PLA_2_ sequences from *C*. *o*. *cerberus*, a subspecies with type I venom, demonstrated that its PLA_2_ clustered within the acidic hemolytic PLA_2_ clade, as is typical of many low toxicity rattlesnake PLA_2_s. Mojave and crotoxin-like PLA_2_ clusters for acidic and basic subunits were also separate from the clades that contained Old World viper neurotoxic PLA_2_ complexes (basic and acidic subunits of a heterodimer PLA_2_ from *Vipera nikolskii* and vaspin subunits from *Vipera aspis aspis*), suggesting the possibility of a separate evolutionary origin for Old World and New World neurotoxic heterodimeric PLA_2_ complexes.

Venom 3FTxs and PLA_2_s can have multiple different, active sites, and individual toxins are rarely tested for all possible activities or substrates, so it is difficult to predict protein activities or to determine if misclassifications are occurring with predictive methods based solely on sequences similarities [37]. Nevertheless, sequence similarity clustering did successfully identify crotoxin and Mojave toxin homologs, PLA_2_s that are associated with serious neurotoxic envenomation symptomology, in known and previously uninvestigated venoms. Two 3FTx transcripts were discovered in *Boiga cynodon* venom that were very similar in sequence to the two subunits of the heterodimeric, prey-specific iriditoxin from *B*. *irregularis* venom, indicating the presence of another lizard and bird-specific neurotoxin within the venom of a closely related species. Full-length venom protein transcripts obtained from venom can therefore be used to screen for particular toxins or venom phenotypes.

As more full-length transcripts become available, high throughput methods such as next-generation proteomic (and transcriptomic) characterization of venoms that lack profiles, including most rear-fanged snake species and many understudied front-fanged snake species, will be greatly facilitated. The methods described here provided full-length venom protein transcripts from venoms representing the three major families of venomous snakes, making it is possible to determine snake venom genotype-phenotype relationships without the need to sacrifice living snakes. By requiring only venom to obtain venom protein cDNAs, the approaches detailed here will provide access to cDNA-based protein sequences in the absence of living specimens, from commercial and other venom sources, and will facilitate study of snake venom protein composition and evolution, and in turn, provide greater predictability of the development of regionally-specific reactions following snakebite envenomation.

## Supporting Information

S1 Text Accession numbers. (DOCX)

S1 Fig **Multiple sequence alignments of the first 75 nucleotides of various Group IIA viperid phospholipase A_2_s (A) and non-conventional three-finger toxins (B).** A) Venom-based PLA_2_ cDNA sequences (asterisks) were obtained from *Crotalus p*. *pricei*, *C*. *cerastes cercobombus*, *C*. *molossus nigrescens*, *C*. *oreganus concolor*, *C*. *oreganus cerberus*, *C*. *basiliscus*, *C*. *simus tzabcan*, and *Sistrurus miliarius barbouri* and were aligned with toxins from several other crotaline species; identical nucleotide sequences are shaded, and regions utilized for a specifically-designed sense primer are indicated by the red bar. This primer sequence includes the end of the 5’UTR and beginning of the signal peptide. GenBank accession numbers for known toxins are as follows: Crotalus_atrox (AF269131), Crotalus_h_horridus (GQ168368.1), Sistrurus_c_tergeminus (AY508692.1), Agkistrodon_contortrix (ACU21335), Lachesis_muta (KM459520.1), Bothriechis_schlegelii (AY764137.1), Vipera_b_berus (AJ580215.1), Echis_carinatus (AY268946.2), Daboia_russellii (DQ090661.1), Gloydius_intermedius (KJ654336.1), Deinagkistrodon_acutus (X77649.1), and Protobothrops_mucrosquamatus (AF408409). B) Venom based 3FTx cDNA sequences (asterisks) were obtained from *Boiga irregularis*, *B*. *dendrophila*, *B*. *nigriceps*, *B*. *cynodon*, *Oxybelis fulgidus*, *Ahaetulla prasina*, and *Trimorphodon biscutatus lambda* and were aligned with toxins from several other rear-fanged and Elapidae species; identical nucleotide sequences are shaded and regions utilized for a specifically-designed sense primer are indicated by the red bar. This primer sequence includes the beginning of the signal peptide. GenBank accession numbers are as follows: Trimorphodon_biscutatus_Tri3 (EU029678.1), Trimorphodon_biscutatus_Tri2 (EU029677.1), Telescopus_dhara_Tel4 (EU029686.1), Boiga_dendrophila_denmo (DQ366293.1), Boiga_irregularis_irditoxinB (DQ304539.1), Boiga_irregularis_irditoxinA (DQ304538.1), Boiga_irregularis_1f (GBSH01000015.1), Thrasop_jacksoni_Thr3 (EU029685.1), Dispholidus_typus_Dis1 (EU029674.1), Telescopus_dhara_Tel1 (EU029675.1), Thrasops_jacksoni_Thr5 (EU036635.1), Trimorphodon_biscutatus_Tri1 (EU029675.1), Naja_atra (AF031472.1), Bungarus_multicinctus (AF056400.1), Ophiophagus_hannah (FJ952515.1), Psammophis_mossambicus_Psa1 (EU029669.1), Leioheterodon_madagascariensis (EU029676.1), Bungarus_candidus (AY057878.1), and Dendroaspis_angusticeps (AF241871.1).
(TIF)

S2 Fig Additional amplified non-toxin transcripts within venom found during primer optimization. Transcript sequences for non-toxin proteins (ribosomal and cathelicidin proteins) were found within both rattlesnake and rear-fanged snake venoms demonstrating that other complete transcripts are found in venoms.
(DOCX)
