# Supplementary material for: Full-Length Venom Protein cDNA Sequences from Venom-Derived mRNA: Exploring Compositional Variation and Adaptive Multigene Evolution
Source: PLoS Negl Trop Dis. 2016 Jun 9;10(6):e0004587. doi: 10.1371/journal.pntd.0004587 (PMC4900637; doi:10.1371/journal.pntd.0004587)
Supplement: S1 Text — (DOCX) [file pntd.0004587.s001.docx]

KU666900: Seq1 [organism=Crotalus cerastes] PLA2 mRNA, complete cds

KU666901: Seq2 [organism=Crotalus simus tzabcan] serine protease_1 mRNA, complete cds

KU666902: Seq3 [organism=Crotalus simus tzabcan] serine protease_2 mRNA, complete cds

KU666903: Seq4 [organism=Crotalus simus tzabcan] serine protease_4 mRNA, complete cds

KU666904: Seq5 [organism=Crotalus simus tzabcan] PLA2_1 Crotoxin-like acidic A chain mRNA, complete cds

KU666905: Seq6 [organism=Crotalus simus tzabcan] PLA2_2 mRNA, complete cds

KU666906: Seq7 [organism=Crotalus simus tzabcan] PLA2_3 mRNA, complete cds

KU666907: Seq8 [organism=Crotalus simus tzabcan] PLA2_4 Mojave toxin-like acidic A chain mRNA, complete cds

KU666908: Seq9 [organism=Crotalus simus tzabcan] C-type lectin_1 mRNA, complete cds

KU666909: Seq10 [organism=Crotalus simus tzabcan] C-type lectin_2 mRNA, complete cds

KU666910: Seq11 [organism=Crotalus simus tzabcan] C-type lectin_3 mRNA, complete cds

KU666911: Seq12 [organism=Crotalus simus tzabcan] C-type lectin_4 mRNA, complete cds

KU666912: Seq13 [organism=Crotalus pricei] PLA2 mRNA, complete cds

KU666913: Seq14 [organism=Crotalus molossus nigrescens] PLA2_1 mRNA, complete cds

KU666914: Seq15 [organism=Crotalus molossus nigrescens] PLA2_2 mRNA, complete cds

KU666915: Seq16 [organism=Crotalus molossus nigrescens] PLA2_3 mRNA, complete cds

KU666916: Seq17 [organism=Crotalus oreganus concolor] PLA2_1 Mojave toxin-like acidic A chain mRNA, complete cds

KU666917: Seq18 [organism=Crotalus oreganus concolor] PLA2_2 Mojave toxin-like basic B chain mRNA, complete cds

KU666918: Seq19 [organism=Crotalus oreganus concolor] PLA2_3 mRNA, complete cds

KU666919: Seq20 [organism=Crotalus oreganus cerberus] PLA2_1 mRNA, complete cds

KU666920: Seq21 [organism=Crotalus oreganus cerberus] PLA2_2 mRNA, complete cds

KU666921: Seq22 [organism=Crotalus basiliscus] PLA2_1 mRNA, complete cds

KU666922: Seq23 [organism=Crotalus basiliscus] PLA2_2 mRNA, complete cds

KU666923: Seq24 [organism=Crotalus basiliscus] PLA2_3 Crotoxin-like basic B subunit mRNA, complete cds

KU666924: Seq25 [organism=Oxybelis fulgidus] 3FTx mRNA, complete cds

KU666925: Seq26 [organism=Ahaetulla prasina] 3FTx_1 mRNA, complete cds

KU666926: Seq27 [organism=Ahaetulla prasina] 3FTx_2 mRNA, complete cds

KU666927: Seq28 [organism=Boiga cynodon] 3FTx_1 mRNA, complete cds

KU666928: Seq29 [organism=Boiga cynodon] 3FTx_2 mRNA, complete cds

KU666929: Seq30 [organism=Boiga irregularis] 3FTx_1 mRNA, complete cds

KU666930: Seq31 [organism=Boiga irregularis] 3FTx_3 mRNA, complete cds

KU666931: Seq32 [organism=Boiga irregularis] 3FTx_4 mRNA, complete cds

KU666932: Seq33 [organism=Boiga irregularis] 3FTx_5 mRNA, complete cds

KU666933: Seq34 [organism=Boiga irregularis] 3FTx_6 mRNA, complete cds

KU666934: Seq35 [organism=Boiga nigriceps] 3FTx mRNA, complete cds

KU666935: Seq36 [organism=Trimorphodon biscutatus lambda] 3FTx_1 mRNA, complete cds

KU666936: Seq37 [organism=Trimorphodon biscutatus lambda] 3FTx_2 mRNA, complete cds

KU666937: Seq38 [organism=Trimorphodon biscutatus lambda] 3FTx_3 mRNA, complete cds
