# Supplementary figures and images for: Full-Length Venom Protein cDNA Sequences from Venom-Derived mRNA: Exploring Compositional Variation and Adaptive Multigene Evolution
Source: PLoS Negl Trop Dis. 2016 Jun 9;10(6):e0004587. doi: 10.1371/journal.pntd.0004587 (PMC4900637; doi:10.1371/journal.pntd.0004587)

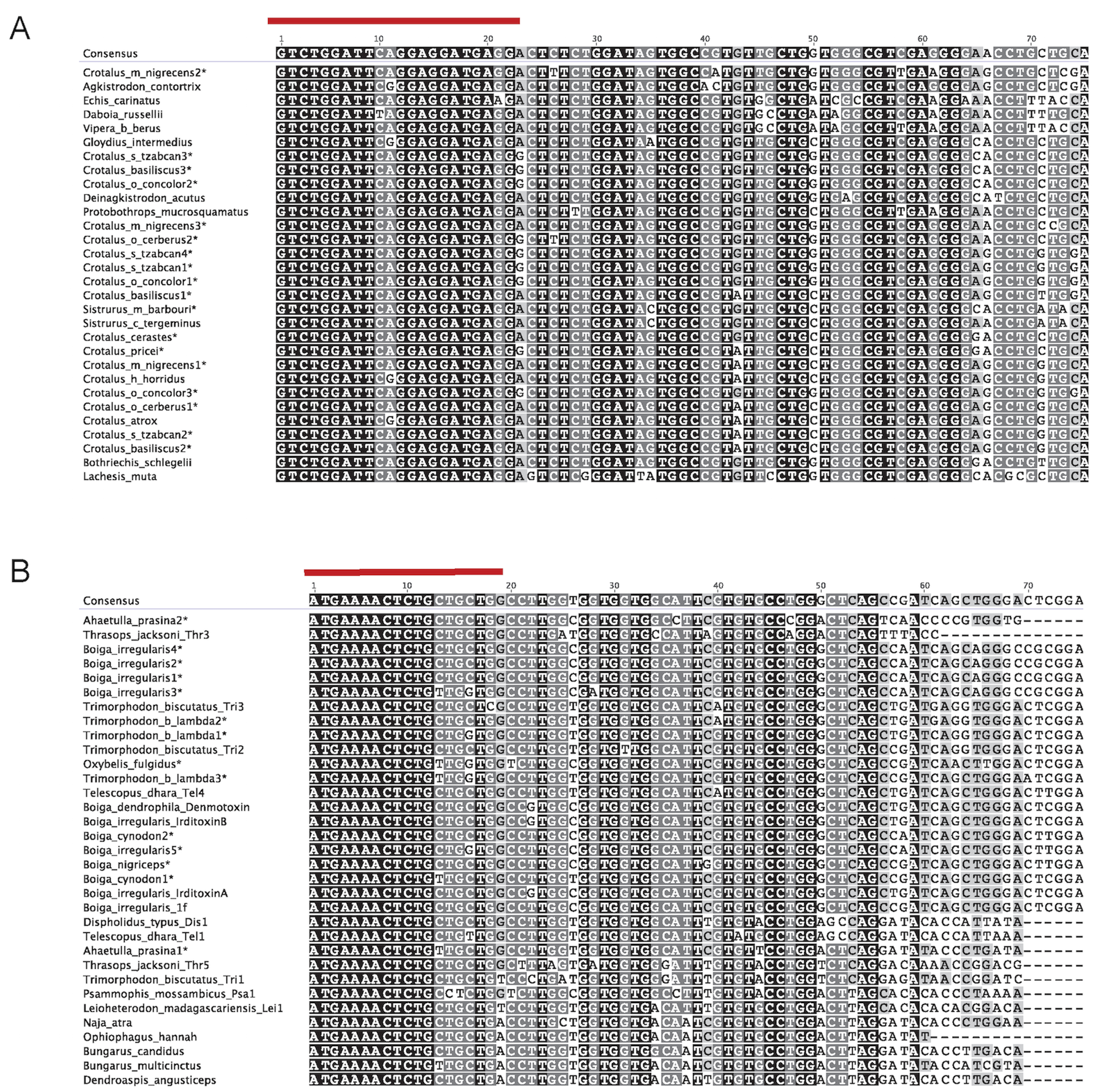

Supplement: S1 Fig — Multiple sequence alignments of the first 75 nucleotides of various Group IIA viperid phospholipase A2s (A) and non-conventional three-finger toxins (B). A) Venom-based PLA2 cDNA sequences (asterisks) were obtained from Crotalus p. pricei, C. cerastes cercobombus, C. molossus nigrescens, C. oreganus concolor, C. oreganus cerberus, C. basiliscus, C. simus tzabcan, and Sistrurus miliarius barbouri and were aligned with toxins from several other crotaline species; identical nucleotide sequences are shaded, and regions utilized for a specifically-designed sense primer are indicated by the red bar. This primer sequence includes the end of the 5’UTR and beginning of the signal peptide. GenBank accession numbers for known toxins are as follows: Crotalus_atrox (AF269131), Crotalus_h_horridus (GQ168368.1), Sistrurus_c_tergeminus (AY508692.1), Agkistrodon_contortrix (ACU21335), Lachesis_muta (KM459520.1), Bothriechis_schlegelii (AY764137.1), Vipera_b_berus (AJ580215.1), Echis_carinatus (AY268946.2), Daboia_russellii (DQ090661.1), Gloydius_intermedius (KJ654336.1), Deinagkistrodon_acutus (X77649.1), and Protobothrops_mucrosquamatus (AF408409). B) Venom based 3FTx cDNA sequences (asterisks) were obtained from Boiga irregularis, B. dendrophila, B. nigriceps, B. cynodon, Oxybelis fulgidus, Ahaetulla prasina, and Trimorphodon biscutatus lambda and were aligned with toxins from several other rear-fanged and Elapidae species; identical nucleotide sequences are shaded and regions utilized for a specifically-designed sense primer are indicated by the red bar. This primer sequence includes the beginning of the signal peptide. GenBank accession numbers are as follows: Trimorphodon_biscutatus_Tri3 (EU029678.1), Trimorphodon_biscutatus_Tri2 (EU029677.1), Telescopus_dhara_Tel4 (EU029686.1), Boiga_dendrophila_denmo (DQ366293.1), Boiga_irregularis_irditoxinB (DQ304539.1), Boiga_irregularis_irditoxinA (DQ304538.1), Boiga_irregularis_1f (GBSH01000015.1), Thrasop_jacksoni_Thr3 (EU029685.1), Dis [file pntd.0004587.s002.tif]
