# Supplementary material for: Full-Length Venom Protein cDNA Sequences from Venom-Derived mRNA: Exploring Compositional Variation and Adaptive Multigene Evolution
Source: PLoS Negl Trop Dis. 2016 Jun 9;10(6):e0004587. doi: 10.1371/journal.pntd.0004587 (PMC4900637; doi:10.1371/journal.pntd.0004587)
Supplement: S2 Fig — Transcript sequences for non-toxin proteins (ribosomal and cathelicidin proteins) were found within both rattlesnake and rear-fanged snake venoms demonstrating that other complete transcripts are found in venoms. (DOCX) [file pntd.0004587.s003.docx]

> *Crotalus cerastes* 60S ribosomal protein: Similar to: PREDICTED: 60S ribosomal protein L7a [Python bivittatus] Sequence ID: ref|XP_007420634.1|

MPKGKKAKGKKVAPAPAVVKKQEAKKVVNPLFEKRPKNFGIGQDIQPKRDLTRFVKWPRYIRLQRQRAILYKRLKVPPAVNQFTQALDRQTATQLLKLAHKYRPETKQEKKQRLLARAEQKAAGKGDVPTKRLPVLRAGVNTVTTLVENKKAQLVVIAHDVDPIELVVFLPALCRKMGVPYCIIKGKARLGRLVXRKTCTCVAFTQVNPEDKGALAKLVEAVKXNYNERYDXIRRHWGGNVL

>*Alsophis portoricensis* 60S ribosomal protein: Similar to: PREDICTED: 60S ribosomal protein L15 isoform X1 [Python bivittatus] Sequence ID: ref|XP_007421748.1|

MGAYKYIQELWRKKQSDVMRFLLRVRCWQYRQLSALHRAPRPTRPDKARRLGYKAKQGYVIYRIRVRRGGRKRPVPKGATYGKPVHHGVNQLKFARSLQSVAEERAGRHCGALRVLNSYWVGEDSTYKFFEVILIDPFHKAIRRNPDTQWITKPVHKHREMRGLTSAXRKXRGLGKGHKFHHTIGGSRRAAXXRHNTLXLHRYR

>*Crotalus scutulatus scutulatus* 40S ribosomal protein: Similar to: PREDICTED: 40S ribosomal protein S9-like isoform X1 [Python bivittatus] Sequence ID: ref|XP_007439934.1|

MPVARSWVCRKTYVTPRRPFEKSRLDQELKLIGEYGLRNKREVWRVKFTLAKIRKAARELLTLDEKDQRRLFEGNALLRRLVRIGVLDEGKMKLDYILGLKIEDFLERRLQTQVFKLGLAKSIHHARVLIRQRHIRVRKQVVNIPSFIVRLDSQKHIDFSLRSPYGGGRPGRVKRKNAKKGQGGAGGGDDEEED

>*Crotalus organus cerberus* antimicrobial peptide: Similar to: PREDICTED: cathelicidin-OH antimicrobial peptide-like [Python bivittatus] Sequence ID: ref|XP_007442672.1|

MQTGRGRALLILLGLALSTPGWTAEGESSVDRDALSSAVERYNSNSGLDSAFRMLQVKSQPDGAPSNPDLQKLELILKETTCPNSPDLNPDDCDFKKNGVVKECSGTSSAQPGSPILQIDCDTTSQGNRRVKRNGKVRKFFRKLKKLLPGGGSTIAHAK

>*Boiga irregularis* antimicrobial peptide: Similar to: PREDICTED: cathelicidin-OH antimicrobial peptide-like [Python bivittatus] Sequence ID: ref|XP_007442672.1|

MQTGRGRALLILLGLAXSTPGWTAEGESSVDRDALSSAVERYNSNSGLDSAFRMLQVKSQPDGAPSNPDLQKLELILKETTCPNSPDLNPDDCDFKKNGVVKECSGTSSAQPGSPILQIDCDTTSQGNRRVKRNGKVRKFFRKLKKLLPGGGSTIAHAK
